# Darunavir in experienced patients

**DOI:** 10.1186/1758-2652-13-S4-P27

**Published:** 2010-11-08

**Authors:** G Sterrantino, B Borchi, M Trotta, D Bartolozzi, F Leoncini

**Affiliations:** 1 Azienda Ospedaliero-Universitaria Careggi, Infectious Diseases, Firenze, Italy

## Purpose of the study

This study aims to assess the performance of DRV in clinical practice, as part of salvage therapy strategies.

## Methods

We did retrospective assessment of HIV+ patients who received DRV at our institution, prescribed as part of a salvage regimen since 2006. Liver, metabolic and renal profile were assessed at baseline, after 1 month and every 3 months. 52 HIV1+ patients have been enrolled; mean age was 48 (IQR 44-54) years, male 82%, IDUs 29%, MSM 37%, heterosexuals 33%; 15 patients were HCV or HBV co-infected. All but one were subtype B. Median CD4 nadir was 82 (IQR 27-234). Thirty-one patients had AIDS history. Mean time on ARVs was 15 (IQR14-17) years; major mutations were: 8 for NRTI, 2 for NNRTI and 5 for PI.

## Results

Median follow-up was 104 weeks (IQR 60—139). Two patients died: one following a car accident, the other one due to disseminated Kaposi's sarcoma. Four patients stopped DRV: one lost to follow-up, one developed decompensated diabetes, one rash, one virological failure. Companion drugs with DRV were NRTI (71%), etravirine (14%), maraviroc (33%), raltegravir (RAL) (33%), enfuvirtide (ENF) (33%). Seventeen patients had changes in therapy during follow up, four patients stopped NRTIs, among 13 patients who stopped ENF, 5 replaced with RAL. Mean CD4 and HIV-RNA values at baseline were 251 cells/mm3 and 4.3 log10 copies/ml, respectively; CD4 median monthly increase was 9 (IQR 5.1-14.5) cells/mm3. After 1 month, 49 % had HIV-RNA <50 copies/ml; after 12, 24 and 33 months 91% of patients were still undetectable. No statistically significant modification were seen in transaminases, creatinine, glucose, triglycerides values.

## Conclusions

Darunavir was highly effective and well tolerated in most of patients and showed good metabolic, renal and liver profile in our cohort where the rate of co-infected patients was high.

